# Exploring the Paradoxical Association Between Chest CT Pulmonary Involvement and Olfactory Dysfunction in COVID‐19 Patients: A Prospective Cross‐Sectional Study

**DOI:** 10.1002/hsr2.72777

**Published:** 2026-07-28

**Authors:** Abdolkarim Ghadimi‐Moghadam, Saed Askari, Mohammad Amin Ghatee, Alireza Mortazavi, Saeed Javdansirat, Ali Ghadimi Moghadam, Hassan Vafapour, Zaker Salehi, Seyed Mohammad Javad Mortazavi, Lembit Sihver

**Affiliations:** ^1^ Department of Pediatric Infectious Diseases Yasuj University of Medical Sciences Yasuj Iran; ^2^ Department of Radiology, Shahid Jalil Hospital Yasuj University of Medical Sciences Yasuj Iran; ^3^ Professor Alborzi Clinical Microbiology Research Center Shiraz University of Medical Sciences, Shiraz, Iran; ^4^ College of Medical, Veterinary & Life Sciences The University of Glasgow Scottland UK; ^5^ Department of Infectious Diseases Yasuj University of Medical Sciences Yasuj Iran; ^6^ Faculty of Medicine Szeged University Szeged Hungary; ^7^ Cellular and Molecular Research Center Yasuj University of Medical Sciences Yasuj Iran; ^8^ Department of Medical Physics, School of Medicine Shiraz University of Medical Sciences Shiraz Iran; ^9^ Department of Informatics and Engineering Systems The University of Texas Rio Grande Valley Brownsville USA; ^10^ Department of Physics East Carolina University Greenville USA; ^11^ Royal Military College of Canada Kingston Canada

**Keywords:** COVID‐19 Patients, CT‐Scans, Lungs, Olfactory Dysfunction, Pulmonary Involvement, Radiological Findings

## Abstract

**Background and Aims:**

Olfactory dysfunction (OD) is common in COVID‐19, yet its relationship with pulmonary involvement remains inconsistent. We evaluated the association between CT‐based lung involvement and OD in laboratory‐confirmed COVID‐19.

**Methods:**

In a prospective cross‐sectional study (Yasuj University of Medical Sciences hospitals; December 26, 2021–May 22, 2022), OD was assessed using the CCCRC protocol and pulmonary involvement was categorized using a CT lung involvement score. Associations were examined using chi‐square testing (categorical) and ANOVA where appropriate (α = 0.05).

**Results:**

Among 120 patients (60 male/60 female; mean age 43.17 ± 14.76 years), 71 (59.2%) had hyposmia, and none had complete anosmia. CT pulmonary involvement was present in 40 (33.3%). OD category was significantly associated with the pulmonary involvement category (chi‐square test; *P* value as reported in the Results section).

**Conclusion:**

OD was frequent and showed a statistically significant association with CT‐based pulmonary involvement; however, OD severity alone should not be used as a stand‐alone surrogate for pulmonary involvement. Larger multicenter studies are needed to clarify the observed pattern.

## 1 Background

COVID‐19 presents with a wide clinical spectrum, and objective measures such as chest CT can quantify pulmonary involvement. Olfactory dysfunction (OD) is also frequently reported in COVID‐19, yet published findings conflict regarding whether OD tracks with disease severity. Clarifying this relationship may improve clinical interpretation of OD in routine assessment [1, 2, 3, 4, 5].

Accordingly, we aimed to evaluate the association between CT‐based pulmonary involvement and OD using standardized olfactory testing and a structured CT involvement score. Anosmia, or the loss of smell, has been identified as a potential early indicator of COVID‐19 infection. Although anosmia is not exclusive to COVID‐19 and can result from other respiratory infections, research has suggested that it may serve as a valuable marker for identifying individuals with more severe forms of the disease [6]. Numerous studies have reported that loss of smell (anosmia) and/or loss of taste (ageusia) are common symptoms associated with COVID‐19. Additionally, some studies have indicated that the severity of smell loss may correlate with the severity of COVID‐19. One study published in the International Forum of Allergy & Rhinology in July 2020 analyzed data from 169 patients with confirmed COVID‐19 who reported olfactory symptoms [7]. The findings revealed that patients with severe COVID‐19 were more likely to experience significant smell loss compared to those with mild to moderate cases of the disease [8, 9].

Another study published in the European Archives of Oto‐Rhino‐Laryngology in August 2020 analyzed data from 2581 patients with confirmed COVID‐19 who reported olfactory symptoms. The study found that patients with more severe COVID‐19 symptoms were more likely to experience loss of smell than those with milder symptoms. A subsequent study published in JAMA Otolaryngology‐Head & Neck Surgery in January 2021 examined data from 279 patients with confirmed COVID‐19 who also reported olfactory symptoms. This study revealed that patients with more severe COVID‐19 symptoms were more likely to experience persistent loss of smell compared to those with milder symptoms. Additionally, another study published in the Journal of Internal Medicine in February 2021 analyzed data from 430 patients with confirmed COVID‐19 who reported olfactory symptoms. This research indicated that patients with severe COVID‐19 were more likely to experience prolonged loss of smell than those with mild to moderate COVID‐19 [10].

A multicenter European study investigated olfactory and gustatory dysfunctions in patients with laboratory‐confirmed COVID‐19 infections. A total of 417 patients with mild‐to‐moderate COVID‐19 from 12 European hospitals completed questionnaires assessing their olfactory and gustatory functions. The most prevalent general symptoms included cough, myalgia, and loss of appetite, while facial pain and nasal obstruction were the most common otolaryngological symptoms related to the disease. Notably, 85.6% of patients reported OD, and 88.0% reported gustatory dysfunction. OD manifested before other symptoms in 11.8% of cases. Scores on the Short Version of the Questionnaire of Olfactory Disorders‐Negative Statements (sQOD‐NS) were significantly lower in patients with anosmia compared to those who were normosmic or hyposmic. Among the 18.2% of patients without nasal obstruction or rhinorrhea, 79.7% were either hyposmic or anosmic. The early olfactory recovery rate was 44.0%. Additionally, females were significantly more affected by olfactory and gustatory dysfunctions than males. The study concludes that olfactory and gustatory disorders are prevalent symptoms among European COVID‐19 patients, even in the absence of nasal symptoms, and that sudden anosmia or ageusia should be recognized by the international scientific community as important indicators of COVID‐19 infection.

Overall, these studies suggest a potential link between the severity of smell loss and the severity of COVID‐19. However, further research is necessary to fully understand the relationship between these factors. A systematic review and meta‐analysis of 27 studies published in May 2021 found that the prevalence of anosmia among COVID‐19 patients was 43%, with the majority of cases classified as mild or moderate. Notably, the study also revealed that anosmia was significantly more prevalent in patients with severe or critical COVID‐19 and was associated with an increased risk of hospitalization and a longer duration of hospital stay.

Another study published in The Lancet in October 2020 found that loss of smell was a prevalent symptom among patients with mild to moderate COVID‐19, occurring more frequently in younger individuals and those with less severe disease. However, the study also indicated that anosmia was not always accompanied by other typical COVID‐19 symptoms, such as cough or fever, and that it was not a reliable indicator of disease severity. A more recent study published in the Journal of Internal Medicine in January 2022 reported that loss of smell was a common early symptom in COVID‐19 patients, occurring in 55% of cases [11, 12, 13, 14, 15, 16, 17, 18, 19, 20].

The study also found that anosmia was more prevalent in patients with severe disease and was associated with an increased risk of hospitalization and mortality. Overall, while anosmia may not serve as a definitive marker of COVID‐19 severity, it appears to be a common and potentially useful symptom for identifying individuals at a higher risk of severe disease. The predominant radiological manifestations of COVID‐19 include airspace opacities, such as consolidations and ground‐glass opacities. These opacities are often observed peripherally, bilaterally, and with a predilection for the lower lung fields. Although several semi‐quantitative scales have been proposed for visually assessing the extent of lung involvement on CT scans, as described by Chamorro et al., these methods often suffer from limited precision. Our departments have been actively engaged in COVID‐19 research, with some of our studies receiving significant global recognition. It is well established that COVID‐19 infection can manifest with olfactory and gustatory dysfunction, both as presenting symptoms and as potential long‐term sequelae. However, comparative analyses investigating the diagnostic value of changes in the sense of smell for predicting the extent of pulmonary involvement, as assessed by CT scans, remain scarce. The purpose of the current study is to investigate the complex correlation between pulmonary involvement on CT scans and OD in COVID‐19 patients, a paradox that warrants further exploration [20, 21, 22].

## 2 Methods

### 2.1 Study Design

This prospective cross‐sectional study was conducted across multiple institutions involving patients who had undergone COVID‐19 testing at teaching hospitals affiliated with Yasuj University of Medical Sciences (YUMS). Consecutive patients with laboratory‐confirmed COVID‐19 were prospectively enrolled to investigate the association between CT‐based pulmonary involvement and olfactory function, as assessed using the Connecticut Chemosensory Clinical Research Center (CCCRC) protocol. Analyses were pre‐specified and designed to be primarily descriptive and association‐focused.

### 2.2 Ethical Considerations

The study was conducted in accordance with the ethical standards of YUMS and received approval from the institutional ethics committee (Ethics Committee Code: IR.YUMS.REC.1400.169). Written consent was obtained from all patients who participated in this study.

### 2.3 Patient Selection

The study was conducted from December 26, 2021, to May 22, 2022, and included 120 COVID‐19 patients (60 men and 60 women). The sample size was informed by feasibility and preliminary pilot data (unpublished); no formal a priori power calculation was performed. All patients were confirmed COVID‐19 cases through laboratory testing using RT‐PCR on nasopharyngeal and/or oropharyngeal swabs. Prior to any tests being performed, patients were interviewed by an experienced member of the research team.

### 2.4 Inclusion and Exclusion Criteria

We adhered to the inclusion and exclusion criteria outlined by Gupta et al [21]. for patient selection. The inclusion criteria consisted of patients with laboratory‐confirmed COVID‐19 via RT‐PCR, individuals over the age of 16, and those who provided informed consent. The exclusion criteria included patients who experienced hyposmia (a reduced sense of smell) or anosmia prior to COVID‐19 infection, individuals with a history of any rhinosinusal surgery, patients with neuropsychiatric disorders, and those admitted to the ICU.

### 2.5 Standard Odor Test

To assess OD, we utilized the standard odor test from the CCCRC. This test involved measuring the odor threshold of n‐butanol at seven different concentrations and identifying various substances, including coffee powder, cinnamon powder, baby powder, peanut butter, chocolate powder, soap powder, and eyelash. Additionally, eight distracting substances such as pepper, cigarette smoke, automobile tire, wood, garlic, tomato sauce, sardines, and onion were employed to evaluate odor identification. Seven substances were tested in each nostril, and the final identification score for each nostril was recorded on a scale of 0 to 7.

### 2.6 CT Scan Lung Involvement Score

A CT scan of the lungs was performed, and a single experienced radiologist assessed the degree of lung involvement using a scoring system ranging from 0 to 20 to minimize interindividual variations. We employed the method described by Chung et al., which involved evaluating the degree of involvement in each of the five lung lobes and categorizing it as none (0%), minimal (1%–25%), mild (26%–50%), moderate (51%–75%), or severe (76%–100%). A lobe score of 0 indicated no involvement, 1 indicated minimal involvement, 2 indicated mild involvement, 3 indicated moderate involvement, and 4 indicated severe involvement. The overall severity score for the lungs was calculated by summing the scores of the five lobes, resulting in a possible total score ranging from 0 to 20.

### 2.7 Statistical Analysis

We conducted a statistical analysis using SPSS software, version 22.0 (IBM Corp, NY, USA). We performed descriptive statistics and routine statistical tests, including the Chi‐square test and the Mann–Whitney *U* test. Additionally, we employed logistic regression to investigate the association between smell loss and the severity of COVID‐19, as determined by clinical examinations and CT scan reports. A *p*‐value of less than 0.05, with a 95% confidence interval, was considered statistically significant. Reporting follows STROBE guidance for cross‐sectional studies. For each comparison, we report the statistical test used alongside effect estimates (where applicable) and 95% confidence intervals, consistent with journal reporting guidance.

## 3 Results

This study reports on 120 laboratory‐confirmed COVID‐19 patients who were referred to Shahid Jalili Hospital in Yasuj, Iran. The patient population was evenly divided by gender, with 50% male and 50% female participants. The mean age of the patients was 43.17 years (±14.76), with an age range of 17 to 81 years. The demographic data of the patients who participated in this study are presented in Table 1.

**Table 1:** Demographic data of the patients participated in this study.

Parameter	COVID‐19 patients
Sample size, *n*	120
Age (mean ± SD), years	43.17 ± 14.76
Gender (M/F), *n*	60/60
Smoker (current/previous, or never smoker), *n*	0.6%/99.4%

Table 1 shows the demographic characteristics of the study participants, including sample size (*n* = 120), mean age (43.17 ± 14.76 years), gender distribution (60 males and 60 females), and smoking status (0.6% current/previous smokers, 99.4% never smokers).

The classification of OD in COVID‐19 patients who participated in this study is indicated in Table 2.

**Table 2:** Classification of olfactory dysfunction in covid‐19 patients participated in this study.

Olfactory dysfunction category	COVID‐19 patients *N* (%)
Normosmia	49 (40.8)
Hyposmia/Anosmia	71 (59.2)
Mild hyposmia	40 (33.3)
Moderate hyposmia	26 (21.7)
Severe hyposmia	5 (4.2)
Anosmia	0 (0)
SubTotal	71 (59.2)

**Figure 1:**
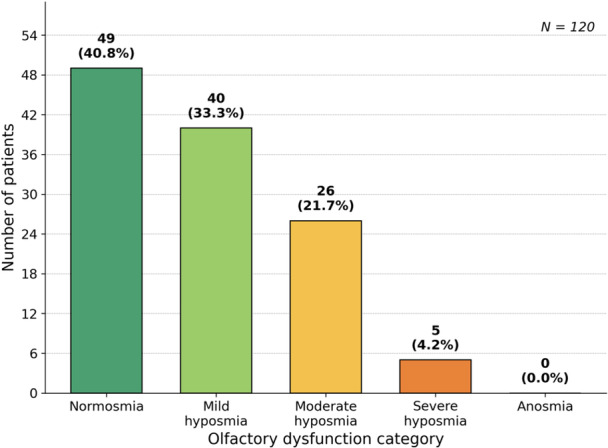
Illustrates the distribution of olfactory dysfunction categories among the 120 COVID‐19 patients.

Table 2 and Figure 1 presents the classification of OD among the COVID‐19 patients in our study. Of the total participants, 49 patients (40.8%) had normosmia, while 71 patients (59.2%) experienced varying degrees of hyposmia. Specifically, 40 patients (33.3%) had mild hyposmia, 26 patients (21.7%) had moderate hyposmia, and 5 patients (4.2%) had severe hyposmia. None of the patients experienced complete anosmia.

**Figure 2:**
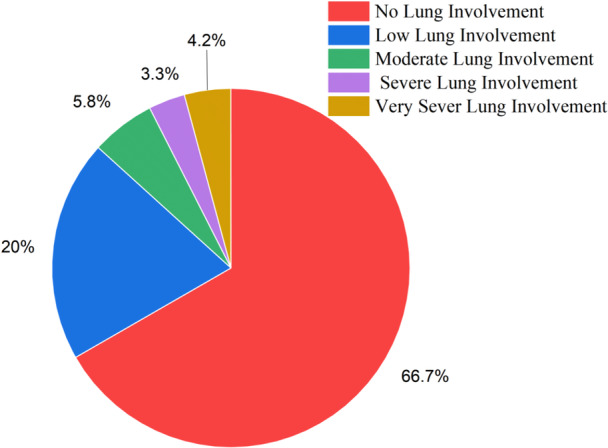
The proportion of different categories of lung involvement in COVID‐19 patients participated in this study.

CT scans were performed on all patients, with 66.7% showing no pulmonary involvement. Mild, moderate, high, and severe pulmonary involvement were observed in 20%, 5.8%, 3.3%, and 4.2% of patients, respectively. The study found no significant relationship between OD and gender (*p* = 0.095) or age (*p* = 0.61). However, younger patients experienced more severe forms of OD (*p* < 0.05). No association was found between gender and the severity of pulmonary involvement on CT scans (*p* = 0.69), but a significant negative correlation was identified between pulmonary involvement and the severity of OD (*p* = 0.02). Additionally, pulmonary involvement was significantly associated with age (*p* < 0.01); younger patients experienced less severe pulmonary involvement. To investigate the relationship between the type and severity of pulmonary involvement in patients based on CT scans, the Exact Fissure test was employed. The results indicated no significant relationship between these variables (*p* = 0.69). Figure 2 illustrates the distribution of various categories of lung involvement among COVID‐19 patients who participated in this study.

Figure 2 displays the proportion of different categories of lung involvement observed in the COVID‐19 patients in our study. The pie chart shows that the majority of patients (66.7%) had no pulmonary involvement, while 20% had mild involvement, 5.8 had moderate involvement, 3.3% had severe involvement, and 4.2% had very severe involvement.

**Figure 3:**
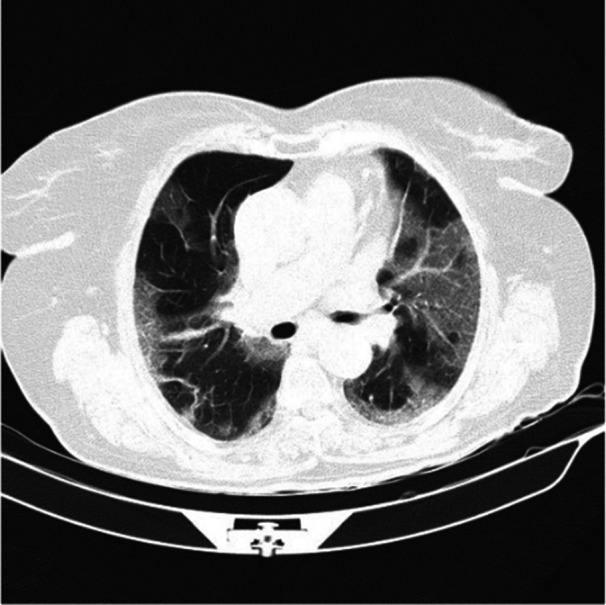
A CT scan section showed severe lung involvement in a 71‐year‐old male/female patient with no history of underlying disease.

One‐way ANOVA analysis revealed a significant relationship between the severity of lung involvement observed in CT scans and the patients’ age (*p* < 0.01). The Tukey HSD post hoc test was employed to identify the specific subgroups contributing to this difference. This analysis indicated significant differences between the severe and mild groups (*p* = 0.002), moderate and mild groups (*p* = 0.007), normal and mild groups (*p* = 0.003), and severe and normal groups (*p* < 0.001). However, no significant difference was found between the severe and moderate involvement groups (*p* = 0.55). The average age across the different groups of lung involvement severity confirmed that patients with more severe lung involvement in chest CT scans tended to be older. Figure 3 illustrates lung involvement in a patient with no prior history of underlying disease.

Figure 3 presents a CT scan image showing severe lung involvement in a 71‐year‐old male patient with no previous history of underlying diseases. The image demonstrates extensive bilateral ground‐glass opacities and consolidations characteristic of severe COVID‐19 pneumonia.

The Chi‐Square test was used to investigate the relationship between the intensity of pulmonary involvement based on CT scans and the severity of smell disorder. The results showed a significant relationship between these variables (*p* = 0.02), which was displayed in the corresponding graph. Severe hyposmia was reported in 5 patients, 60% of whom had no pulmonary involvement, and 40% had mild involvement. Among the 26 patients with moderate olfactory disorder, 53.8% had no lung involvement, 30.8% had mild involvement, and 15.4% had very severe involvement. For the 40 patients with mild smell disorder, 47.5% had no lung involvement, 22.5% had mild involvement, and 17.5% had moderate involvement, while 10% had severe and 2.5% had very severe lung involvement. Among the 49 patients with normal olfactory function, 89.8% had no lung involvement, and 10.2% had mild lung involvement. The association between OD and pulmonary involvement is demonstrated in Table 3.

**Table 3:** Distribution of ct pulmonary involvement categories across olfactory dysfunction (od) categories (*n*, row %), *N* = 120.

OD category	CT Normal	CT Mild	CT Moderate	CT Severe	CT Very severe	Row total
Normosmia	44 (89.8%)	5 (10.2%)	0 (0.0%)	0 (0.0%)	0 (0.0%)	49 (100%)
Mild hyposmia	19 (47.5%)	9 (22.5%)	7 (17.5%)	4 (10.0%)	1 (2.5%)	40 (100%)
Moderate hyposmia	14 (53.8%)	8 (30.8%)	0 (0.0%)	0 (0.0%)	4 (15.4%)	26 (100%)
Severe hyposmia	3 (60.0%)	2 (40.0%)	0 (0.0%)	0 (0.0%)	0 (0.0%)	5 (100%)
Column total (of *N* = 120)	80 (66.7%)	24 (20.0%)	7 (5.8%)	4 (3.3%)	5 (4.2%)	120

Table 3 demonstrates the association between OD and pulmonary involvement in our COVID‐19 patients. The graph clearly illustrates the inverse relationship between the severity of OD and the extent of pulmonary involvement, with patients having normal olfactory function showing the lowest percentage of pulmonary involvement (89.8% with normal CT findings).

## 4 Discussion

Olfactory dysfunction is often one of the earliest symptoms of COVID‐19 and can occur even in the absence of other symptoms. OD has been proposed as a predictive factor for severe disease. Our study is in line with those that provide evidence of a paradox regarding the role of OD in the severity of COVID‐19. Like several other studies, our study found an inverse correlation between disease severity and prevalence of OD. Our study further confirms substantial evidence that indicates OD is a common symptom of COVID‐19. Moreover, in line with other studies that show paradoxical findings, our results indicate that patients with OD, as an initial symptom, may have milder disease compared to those who develop other symptoms. These findings reveal that OD could serve as an early warning sign of a less severe course of the disease. Table 4 provides a summary of the similarities and differences between our study and the study conducted by Alcas et al. [6].

**Table 4:** The similarities and differences between our study and the study conducted by alcas et al. are summarized for easy comparison.

Parameter	Present study (our findings)	Alcas et al. [6]
Study design	Observational, cross‐sectional	Observational, prospective cohort
Sample size (*n*)	120	129
Sex (Male/Female)	60/60 (50.0%/50.0%)	72.1%/27.9%
Age (mean ± SD), years	43.17 ± 14.76	61.6 ± 15.5
Relative frequency of OD	71/120 (59.2%)	24%
Association between age and OD	Not significant (*p* = 0.61)	Inverse (*p* = 0.002)
Association between sex and OD	Not significant (*p* = 0.095)	Not reported
Association between COVID‐19 severity and OD	Significant association (Chi‐square *p* = 0.02)	Not significant (*p* = 0.056)
Association between COVID‐19 severity and age	Statistically significant (*p* < 0.01)	Statistically significant (*p* = 0.003)
Association between COVID‐19 severity and cough	Not assessed	Statistically significant (*p* < 0.001)
Association between COVID‐19 severity and respiratory distress	Not assessed	Statistically significant (*p* = 0.003)

*Note:* “Not assessed” indicates variables not collected/analyzed in the present study. “Not reported” indicates information not reported in Alcas et al. Severity definitions may differ between studies; the present study operationalized severity using CT pulmonary involvement categories.

Abbreviation: OD, olfactory dysfunction.

Table 4 compares our findings with those of Alcas et al., highlighting similarities and differences between the two studies. Key comparisons include study design (our observational, cross‐sectional study *vs.* their observational, prospective cohort study), patient demographics (our study had 120 patients with equal gender distribution *vs.* their 129 patients with 72.1% males), mean age (43.17 ± 14.76 years in our study *vs.* 61.6 ± 15.5 years in theirs), and various statistical associations between OD, COVID‐19 severity, age, and gender. A key limitation is that CT lung involvement scoring was performed by a single radiologist without a formal inter‐reader reliability assessment; this may limit reproducibility and introduce potential measurement bias.

We believe that the observed negative relationship between OD and the severity of pulmonary involvement in our COVID‐19 patients may not necessarily be a paradox, but rather a complex interplay of multiple factors related to the disease process and host response. Although the reason for the inverse correlation between COVID‐19 severity and prevalence of OD is not yet fully understood, several potential reasons could explain this phenomenon:

**Viral Load**: One possible explanation is that the severity of COVID‐19 symptoms, including OD, may be associated with the viral load or the amount of the virus present in the body. It has been hypothesized that individuals with higher viral loads may experience more severe symptoms, including loss of smell or taste. It's possible that individuals with higher viral loads may have a higher prevalence of OD, while those with lower viral loads may have less severe or no OD.

**Immune Response**: Another possibility is that the immune response to the virus could play a role in the severity of OD. Some research suggests that a strong immune response to the virus may help to clear the virus more quickly, resulting in less severe symptoms. On the other hand, a weak or dysregulated immune response may lead to a higher viral load and more severe symptoms, including OD.

**Host Factors**: Individual factors such as genetics, age, and overall health status may also influence the severity of COVID‐19 symptoms, including OD. For example, older individuals or those with pre‐existing health conditions may be more susceptible to severe COVID‐19 symptoms, while younger, healthier individuals may experience milder symptoms, including less severe OD.

**Variability in Reporting and Testing**: There may also be variability in reporting and testing of OD in different populations, which could impact the observed prevalence rates. Differences in testing methods, availability of testing, and reporting practices in different regions or countries could contribute to the observed inverse correlation between COVID‐19 severity and prevalence of OD. Our study revealed an unexpected inverse relationship between the severity of OD and the extent of pulmonary involvement in COVID‐19 patients. This seemingly paradoxical finding suggests that patients with minimal or absent olfactory symptoms may be at increased risk of severe lung disease. One possible explanation for this observation could be a differential impact of the virus on different organ systems, with more severe infections potentially overwhelming the olfactory epithelium while simultaneously causing significant respiratory complications.

## 5 Conclusion

In this prospective cross‐sectional cohort, OD was common and showed a statistically significant association with CT‐based pulmonary involvement. Given the observational design and the non‐uniform pattern across OD categories, OD should be interpreted as a clinical feature that may co‐occur with pulmonary involvement rather than a reliable proxy for respiratory disease burden. Prospective multicenter studies with standardized timing of symptom assessment and imaging are warranted [11, 12, 13, 14, 15].

## Author Contributions

**Abdolkarim Ghadimi‐Moghadam:** writing – original draft, supervision, resources. **Saed Askari:** investigation, software. **Mohammad Amin Ghatee:** validation, visualization, data curation. **Alireza Mortazavi:** formal analysis, resources. **Saeed Javdansirat:** validation. **Ali Ghadimi Moghadam:** formal analysis. **Hassan Vafapour:** data curation, formal analysis, software, writing – original draft, writing – review and editing, project administration. **Zaker Salehi:** validation, funding acquisition. **Seyed Mohammad Javad Mortazavi:** supervision, methodology, conceptualization, resources.

## Funding

The authors have nothing to report.

## Consent

Written consent was obtained from all patients who participated in this study.

## Conflicts of Interest

The authors declare no conflicts of interest.

## Transparency Statement

Corresponding author affirms that this manuscript is an honest, accurate, and transparent account of the study being reported; that no important aspects of the study have been omitted; and that any discrepancies from the study as planned (and, if relevant, registered) have been explained.

## Data Availability

The data that support the findings of this study are available on request from the corresponding author. The data are not publicly available due to privacy or ethical restrictions.

## References

[1] B. A. Othman , S. Q. Maulud , P. J. Jalal , et al., “Olfactory Dysfunction as a Post‐Infectious Symptom of SARS‐CoV‐2 Infection,” Annals of Medicine & Surgery 75 (2022): 103352. 35169465 10.1016/j.amsu.2022.103352 PMC8830927

[2] K. Karamali , M. Elliott , and C. Hopkins , “COVID‐19–Related Olfactory Dysfunction,” Current Opinion in Otolaryngology & Head and Neck Surgery 30, no. 1 (2022): 19–25. 34889850 10.1097/MOO.0000000000000783 PMC8711304

[3] R. Butowt , K. Bilinska , and C. S. von Bartheld , “Olfactory Dysfunction in COVID‐19: New Insights into the Underlying Mechanisms,” Trends in Neurosciences 46, no. 1 (2023): 75–90. 36470705 10.1016/j.tins.2022.11.003 PMC9666374

[4] Y. Mao , B. Ye , C. Fan , et al., “Correlation Between Coronavirus Disease 2019 and Olfactory Dysfunction,” Frontiers in Public Health 10 (2022): 843850. 35392472 10.3389/fpubh.2022.843850 PMC8980590

[5] B. Prem , D. T. Liu , G. Besser , et al., “Long‐Lasting Olfactory Dysfunction in COVID‐19 Patients,” European Archives of Oto‐Rhino‐Laryngology 279, no. 7 (2022): 3485–3492. 34757458 10.1007/s00405-021-07153-1 PMC8578909

[6] O. Alcas , D. Saldaña , A. Triveño , M. Salazar , and P. M , “Association Between Olfactory Dysfunction and COVID‐19 Severity: A Prospective Study in a Highly Complex Hospital in Peru,” Ear, Nose, & Throat Journal 100, no. 10_suppl (2021): 586S–592S. 10.1177/01455613211066691 34908507

[7] V. Gupta , L. Banavara Rajanna , K. Upadhyay , et al., “Olfactory and Gustatory Dysfunction in COVID‐19 Patients From Northern India: A Cross‐Sectional Observational Study,” Indian Journal of Otolaryngology and Head and Neck Surgery: Official Publication of the Association of Otolaryngologists of India 73, no. 2 (2021): 218–225. 33589874 10.1007/s12070-021-02391-5 PMC7875162

[8] M. Chung , A. Bernheim , X. Mei , et al., “CT Imaging Features of 2019 Novel Coronavirus (2019‐nCoV),” Radiology 295, no. 1 (2020): 202–207. 32017661 10.1148/radiol.2020200230 PMC7194022

[9] J. J. Bevelacqua , S. A. R. Mortazavi , and S. M. J. Mortazavi , “Re: Low‐Dose Radiation Therapy for COVID‐19 Pneumonia: Is There Any Supportive Evidence?,” International Journal of Radiation Biology 96, no. 10 (2020): 1236–1237. 32673140 10.1080/09553002.2020.1797213

[10] M. Ghaderian , M. Kiani , S. Shahbazi‐Gahrouei , D. Shahbazi‐Gahrouei , A. Ghadimi Moghadam , and M. Haghani , “COVID‐19 and MERS: Are Their Chest X‐Ray and Computed Tomography Scanning Signs Related?,” Journal of Medical Signals & Sensors 12, no. 1 (2022): 1–6. 35265460 10.4103/jmss.JMSS_84_20 PMC8804588

[11] D. Firoozi , M. Haqqani , S. Javadan Sirat , A. Paymard , and A. Ghadimi Moghadam , “Investigation of the Prevalence of Underlying Diseases in COVID‐19 Patients in Yasuj,” Armaghane Danesh 25 (2021): 937–944.

[12] S. A. Mortazavi , J. J. Bevelacqua , P. Rafiepour , et al., “Revisiting the Paradox of Smoking: Radioactivity in Tobacco Smoke or Suppressing the SARS‐CoV‐2 Receptor ACE2 via Aryl‐Hydrocarbon Receptor Signaling?,” Dose‐Response 20, no. 1 (2022): 15593258221075111. 35392263 10.1177/15593258221075111 PMC8980405

[13] A. Mortazavi , S. M. J. Mortazavi , and L. Sihver , “Selective Pressure‐Free Treatments for COVID‐19,” Radiation 1, no. 1 (2020): 18–32.

[14] S. M. J. Mortazavi , S. F. Shams , S. Mohammadi , S. A. R. Mortazavi , and L. Sihver , “Low‐Dose Radiation Therapy for COVID‐19: A Systematic Review,” Radiation 1, no. 3 (2021): 234–249.

[15] S. M. J. Mortazavi , B. B. B. Zarandi , A. Jafarzadeh , S. A. Mortazavi , and L. Sihver , “COVID‐19 Update: The Golden Time Window for Pharmacological Treatments and Low‐Dose,” Radiation 2, no. 3 (2022): 268–272.

[16] R. Yarbakhsh , S. A. R. Mortazavi , S. Mortazavi , H. Parsaei , and D. Rad , “Artificial Intelligence Effectively Predicts the COVID‐19 Death Rate in Different UK Cities,” Journal of Intelligent & Fuzzy Systems 43, no. 2 (2022): 1853–1857.

[17] S. J. Masoumi , M. Haghani , P. Mokkaram , et al., “Family History of Alzheimer's Disease Increases the Risk of COVID‐19 Positivity: A SUMS Employees Cohort‐Based Study,” Journal of Biomedical Physics and Engineering 13, no. 4 (2023): 363–366. 37609510 10.31661/jbpe.v0i0.2104-1318 PMC10440408

[18] A. R. Mehdizadeh , J. J. Bevelacqua , S. A. R. Mortazavi , J. S. Welsh , and S. M. J. Mortazavi , “How Antivirals Might Be Linked to the Emergence of New Variants of SARS‐CoV‐2,” Journal of Biomedical Physics & Engineering 11, no. 2 (2021): 123–124. 33937119 10.31661/jbpe.v0i0.2101-1275 PMC8064135

[19] S. A. Mortazavi , “Coming Out of Nowhere: The Paradox of the Birth of Omicron,” Journal of Biomedical Physics and Engineering 12, no. 4 (2022): 325–326. 36059290 10.31661/jbpe.v0i0.2202-1456 PMC9395623

[20] A. R. Mehdizadeh , J. J. Bevelacqua , J. S. Welsh , S. A. R. Mortazavi , L. Haghshenas , and S. M. J. Mortazavi , “Why Are Physicists Involved in the Studies on the Origin of SARS‐CoV‐2?,” Journal of Biomedical Physics & Engineering 11, no. 4 (2021): 413–414. 34458188 10.31661/jbpe.v0i0.2106-1361 PMC8385222

[21] J. J. Bevelacqua , S. A. R. Mortazavi , J. S. Welsh , and S. M. J. Mortazavi , “How Reactivation of SARS‐CoV‐2 in Astronauts With Dysregulated Immune Systems Can Affect Future Space Missions,” Journal of Biomedical Physics and Engineering 13, no. 3 (2023): 297–298. 37312889 10.31661/jbpe.v0i0.2104-1321 PMC10258206

[22] M. Karimpour , M. Haghani , J. J. Bevelacqua , et al., “The Paradoxical Role of Far‐Ultraviolet C (Far‐UVC) in Inactivation of SARS‐CoV‐2: The Issue of Droplet Size,” Journal of Biomedical Physics & Engineering 12, no. 5 (2022): 535–538. 36313407 10.31661/jbpe.v0i0.2204-1482 PMC9589074

